# Glycerosome of *Melissa officinalis* L. Essential Oil for Effective Anti-HSV Type 1

**DOI:** 10.3390/molecules25143111

**Published:** 2020-07-08

**Authors:** Giulia Vanti, Sotirios G. Ntallis, Christos A. Panagiotidis, Virginia Dourdouni, Christina Patsoura, Maria Camilla Bergonzi, Diamanto Lazari, Anna Rita Bilia

**Affiliations:** 1 Department of Chemistry, University of Florence, Via Ugo Schiff 6, 50019 Sesto Fiorentino (FI), Italy; giulia.vanti@unifi.it (G.V.); mc.bergonzi@unifi.it (M.C.B.); 2 Department of Pharmacognosy/Pharmacology, School of Pharmacy, Aristotle University of Thessaloniki, 54124 Thessaloniki, Greece; sotirios.ntallis@gmail.com (S.G.N.); pchristo@pharm.auth.gr (C.A.P.); virgdour@hotmail.com (V.D.); chripats@pharm.auth.gr (C.P.); dlazari@pharm.auth.gr (D.L.)

**Keywords:** *Melissa officinalis* essential oil, GC-MS, drug delivery, nanovesicles, glycerosomes, stability studies, in vitro release, anti HSV-1 activity, luciferase assay

## Abstract

Essential oils are complex mixtures of strongly active compounds, very volatile and sensitive to light, oxygen, moisture and temperature. Loading inside nanocarriers can be a strategy to increase their stability and successfully use them in therapy. In the present study, a commercial *Melissa officinalis* L. (Lamiaceae) essential oil (MEO) was analyzed by gas chromatography-mass spectrometry, loaded inside glycerosomes (MEO-GS) and evaluated for its anti-herpetic activity against HSV type 1. MEO-GS analyses were prepared by the thin layer evaporation method and they were characterized by light scattering techniques, determining average diameter, polydispersity index and ζ-potential. By transmission electron microscopy, MEO-GS appeared as small nano-sized vesicles with a spherical shape. MEO encapsulation efficiency inside glycerosomes, in terms of citral and β-caryophyllene, was found to be ca. 63% and 76% respectively, and MEO release from glycerosomes, performed by dialysis bag method, resulted in less than 10% within 24h. In addition, MEO-GS had high chemical and physical stability during 4 months of storage. Finally, MEO-GS were very active in inhibiting HSV type 1 infection of mammalian cells in vitro, without producing cytotoxic effects. Thus, MEO-GS could be a promising tool in order to provide a suitable anti-herpetic formulation.

## 1. Introduction

Herpes labialis is the most frequent clinical manifestation of reactivated herpes simplex virus type 1 (HSV-1) infections. About 80 percent of the global population carries HSV-1. After establishing latency, HSV can reactivate causing frequent infections in some patients. Since 1970, several efficient antiviral drugs have been developed, and in particular, acyclovir, the most commonly used drug in HSV treatment, is able to specifically inhibit the viral DNA polymerase when new viral DNA is synthesized during the replication cycle. Although antiviral therapy by acyclovir and other related nucleoside analogs has allowed continual and substantial progress in the treatment of both primary and recurrent infections, some limitations of these drugs have been reported in recent years, mainly represented by viral resistance and long-term toxicity [1]. Consequently, new antiviral agents are urgently needed to obtain effective antiviral therapies.

In this panorama, natural products have a key role because they still represent a main source of bioactive molecules thanks to their enormous structural and chemical diversity, giving them unique characteristics such as their multi-targeting activities. However, in many cases their clinical use is limited due to stability and bioavailability issues [2,3,4]. In particular, essential oils are widely used in folk medicine and many of them can directly inactivate herpes virus and interfere with virion envelope structures or mask viral structures that are necessary for adsorption or entry into host cells, also inhibiting acyclovir-resistant HSV-1 isolates, as demonstrated by the assays with isolated constituents or some essential oils [5,6,7].

One of the most known antiviral essential oils is the lemon balm essential oil obtained from hydro-distillation of *Melissa officinalis* L. (Lamiaceae) [8]. Previously, some studies have focused on the anti-HSV-1 activity of aqueous or hydro-alcoholic extracts of *Melissa officinalis* and their principal phenolic compounds, namely caffeic acid, *p*-coumaric acid and rosmarinic acid [9,10,11,12].

Other studies were focused on the essential oil and/or isolated main constituents citral and β-caryophillene against HSV-1 and/or HSV-2, but none of them were given using a formulated essential oil [13,14].

In folk medicine, essential oils are applied on the skin only after dilution because of the topical irritation and there is an urgent need to formulate suitable drug delivery systems for topical application. Essential oils are complex mixtures of strongly active compounds, very volatile and sensitive to light, oxygen, moisture and temperature. Accordingly, their loading in nanocarriers can represent a smart strategy to stabilize them, as well as to control their delivery after administration [15]. These nanocarriers are vesicles, micelles, lipid nanoparticles (solid lipid nanoparticles, SLN and nanostructured lipid carrier, NLC), polymeric nanoparticles, micro/nanoemulsions and cyclodextrin complexes [2,3,4]. The present study focused on *Melissa officinalis* essential oil (MEO) loaded in glycerosomes, a special kind of vesicles obtained by adding a high concentration of glycerol (10–30% *v*/*v*). Glycerol is a polyalcohol, largely used as humectant in semisolid preparations, which can increase the fluidity and deformability of the vesicle bilayer, able to improve the permeation through the skin [16]. Finally, the developed MEO-loaded nanovesicles were evaluated for their in vitro activity against HSV-1 [17].

## 2. Results and Discussion

### 2.1. Chemical Analysis of MEO by Gas Chromatography–Mass Spectrometry (GC–MS)

A commercial MEO from Chiron Kentauros was analyzed by GC-MS, in order to evaluate the qualitative and quantitative composition (Table 1).

The obtained results were consistent with the data reported in the literature [8]. Thirty constituents were unambiguously identified, and they represented 97.21% of total MEO. Two monoterpenes, geranial and neral, and a bicyclic sesquiterpene, β-caryophyllene, were found to be the main constituents of MEO, representing 36.73%, 27.31% and 14.85%, respectively, of the total constituents. Indeed, geranial is also called citral A and it is the trans-isomer, while neral is called citral B and it is the cis-isomer.

### 2.2. Vesicle Preparation and Physical Characterization

**Figure 1:**
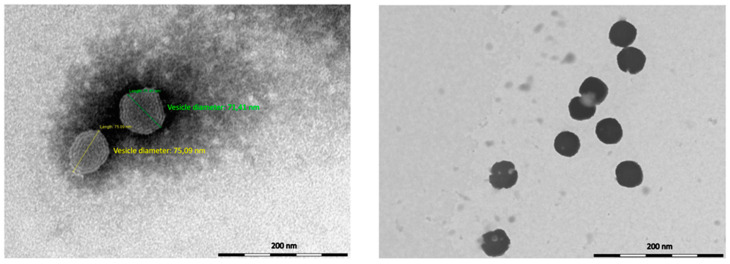
Images of *Melissa officinalis* essential oil-loaded glycerosomes (MEO-GS) obtained by Transmission Electron Microscopic (TEM) analysis.

As a first step of our investigation, MEO was formulated in liposomal vesicles using P90G and cholesterol in different ratios in order to optimize the formulation in terms of vesicle average size and homogeneity. However, after adding 5, 10 or 25 mg/mL of MEO, the vesicles were hardly reproducible and poorly stable, with frequent essential oil separation. Therefore, a slightly different approach was adopted to formulate MEO. Glycerosomes, vesicles containing glycerol, were formulated and loaded with MEO. Briefly, the lipid film, composed of P90G and cholesterol, was hydrated in different conditions using a 10% *v*/*v* glycerol/water solution. MEO (10 mg/mL) was loaded in glycerosomes, optimizing the experimental conditions of preparation, as reported in the experimental section. All the samples were analyzed by light scattering techniques in order to select a homogeneous and stable formulation, with nano-sized vesicles. *Melissa officinalis* essential oil-loaded glycerosomes (MEO-GS), obtained with P90G plus cholesterol (60:1) and loaded with 10 mg/mL of MEO, had small dimensions, low Polydispersity Index (PdI) score and good ζ-potential after the hydration process (Table 2), therefore no additional optimization, for example by ultrasonication probe or extrusion, was necessary. In addition, TEM analysis showed the morphological features of MEO-GS, characterized by spherical shape vesicles with several lamellae (Figure 1).

### 2.3. Encapsulation Efficiency (EE) and Recovery (R) of MEO-GS

Moreover, encapsulation efficiency (EE) and recovery (R) of MEO loaded in GS were evaluated by high performance liquid chromatograph (HPLC) equipped with a diode array detector (DAD) (HPLC-DAD) and not by GC-MS because of the aqueous medium of vesicles. Due to the complexity of the chemical constituents of MEO, marker constituents were chosen in order to evaluate the loading of MEO in glycerosomes. Selection of markers was made on the basis of the most representative constituents in terms of percentage, suitability of UV absorbance for an easy detection by DAD and their availability on the market as standard constituents. Accordingly, both β-caryophyllene (about 15%) and citral (about 64%) were selected as markers. In particular, citral is the mixture of the two geometric monoterpene isomers geranial and neral.

MEO recovery (R) was expressed as percentage of citral and β-caryophyllene recovered after the preparation procedure. Notably, a slight reduction in citral and β-caryophyllene amount occurred during the vesicle preparation, probably due to their high volatility (Table 2). MEO encapsulation efficiency (EE), in terms of citral and β-caryophyllene encapsulated inside glycerosomes, was quite high for both components, mainly considering the respective R percentages (Table 2).

### 2.4. Deformability

MEO loading inside glycerosomes did not modify the fluidity of the vesicle bilayer, maintaining glycerosome ability to squeeze through skin pores. In fact, deformability of MEO-GS was measured by extrusion process, in order to evaluate the ability of vesicles to penetrate the *stratum corneum* passing through the corneocyte pores without breaking and delivering the essential oil to the lower skin layers. MEO-loaded glycerosomes did not change sizes or homogeneity (Table 3) after the extrusion, proving an excellent deformability. This finding suggested that MEO-GS are able to regain their original form after leaving the corneocyte pores, continuing the penetration process [18]. Therefore, the ratio of phospholipid, cholesterol and glycerol was optimal to give flexibility to the bilayer membrane, allowing MEO-GS to pass through pores smaller than their own diameter.

### 2.5. In Vitro Release

**Figure 2:**
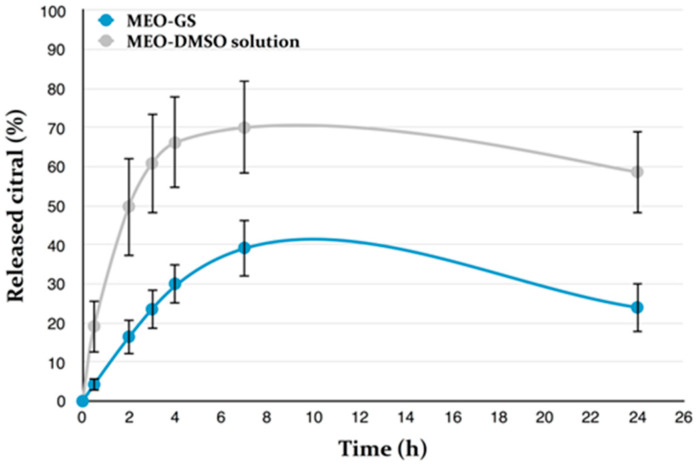
Citral release from *Melissa officinalis* essential oil-loaded glycerosomes (MEO-GS) and MEO- dimethyl sulfoxide (DMSO) solution; (Mean± SD; *n* = 3).

In vitro release profile of MEO from glycerosomes in PBS was investigated using the dialysis bag method and it was compared with the release from a dimethyl sulfoxide (DMSO) solution, as reported in Figure 2. After 24 h, about 24% of citral, selected as marker constituent of MEO, was released from glycerosomes, whereas 59% ca. of citral was released from the DMSO solution. By contrast, β-caryophyllene was never detected. The use of other release mediums, as 20% *v*/*v* ethanol/PBS solution, 10% *v*/*v* glycerol/PBS solution, 5% *v*/*v* DMSO/PBS solution, did not improve β-caryophyllene detection, and PBS was then selected as medium for the release studies. Although the variation of citral percentage within 24 h was comparable between glycerosomes and DMSO solution, with a slow decrease after 7 h probably due to its high volatility, an evident smaller citral release was observed for glycerosomes. Therefore, MEO-GS were able to control and delay MEO release, with a consequent benefit once the formulation will be applied on the skin.

### 2.6. Stability Studies

**Figure 3:**
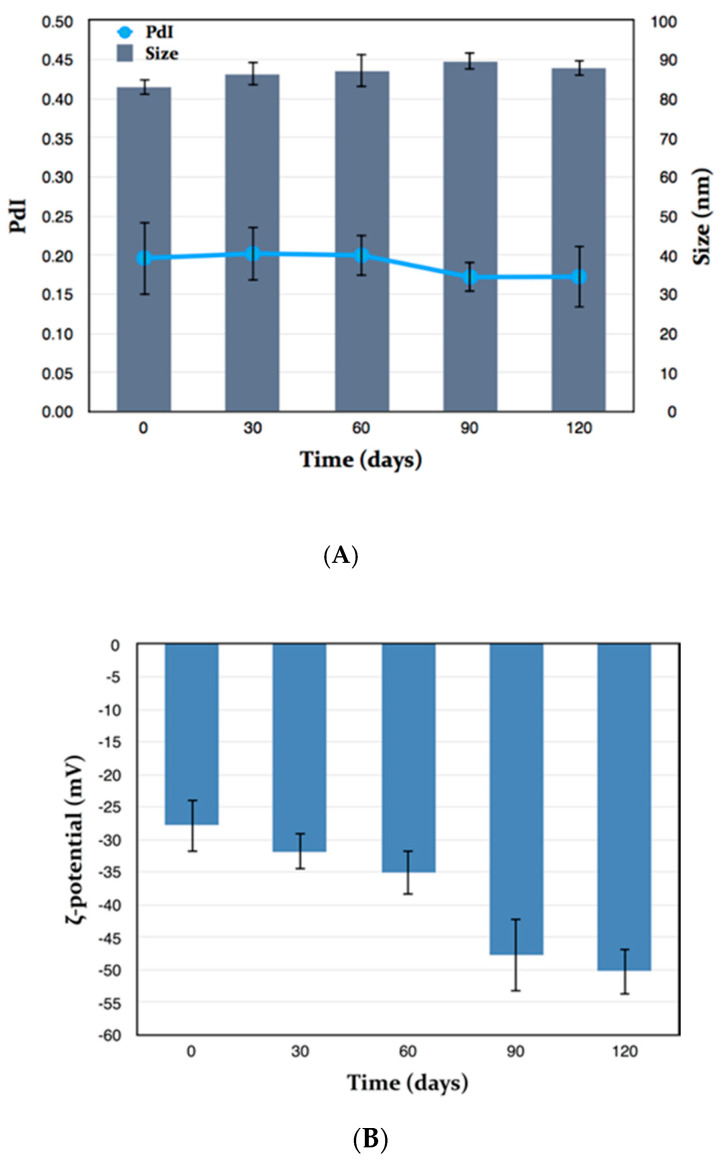
Physical stability of *Melissa officinalis* essential oil-loaded glycerosomes (MEO-GS) during 4 months storage. Size (nm) and PdI (**A**), ζ-potential (mV) (**B**); (Mean ± SD; *n* = 3).

The physical and chemical stability of MEO-GS were investigated during storage of the sample for 4 months at 4 °C, protected from light. Every 30 days, size, PdI and ζ-potential were measured by Dynamic and Electrophoretic Light Scattering (DLS-ELS), whereas R and EE of citral and β-caryophyllene were evaluated by HPLC-DAD. Under the same storage conditions, pure MEO chemical stability was also monitored in terms of citral and β-caryophyllene concentrations. MEO-GS showed excellent physical stability, because sizes and PdI remained unchanged during storage (Figure 3A), whereas the ζ-potential constantly, but slightly, became more and more negative (Figure 3B). This variation was not considered a negative effect because high negative values of charge distribution have positive effects on vesicle stability. Stability of stored MEO in terms of the marker constituents’ citral and β-caryophyllene was also evaluated.

**Figure 4:**
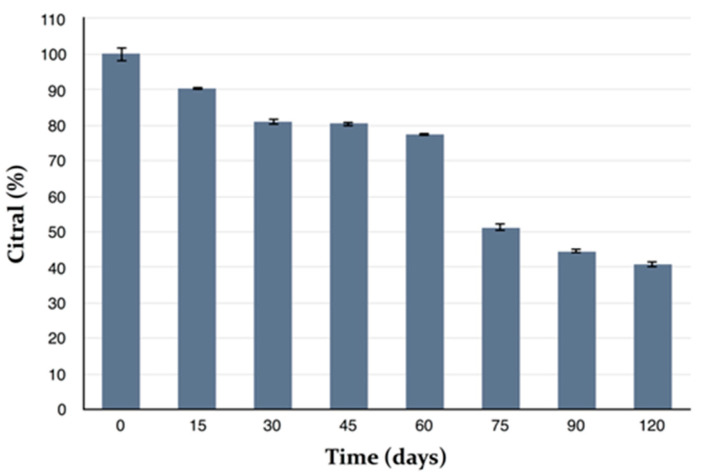
Chemical stability of *Melissa officinalis* essential oil (MEO), in terms of citral concentration, during 4 months storage; (Mean ± SD; *n* = 3).

Pure MEO showed a very low stability of constituents. β-Caryophyllene was detectable in trace amounts after one month of storage. Citral content greatly decreased during storage, and about 59% of citral was lost after 4 months (Figure 4).

**Figure 5:**
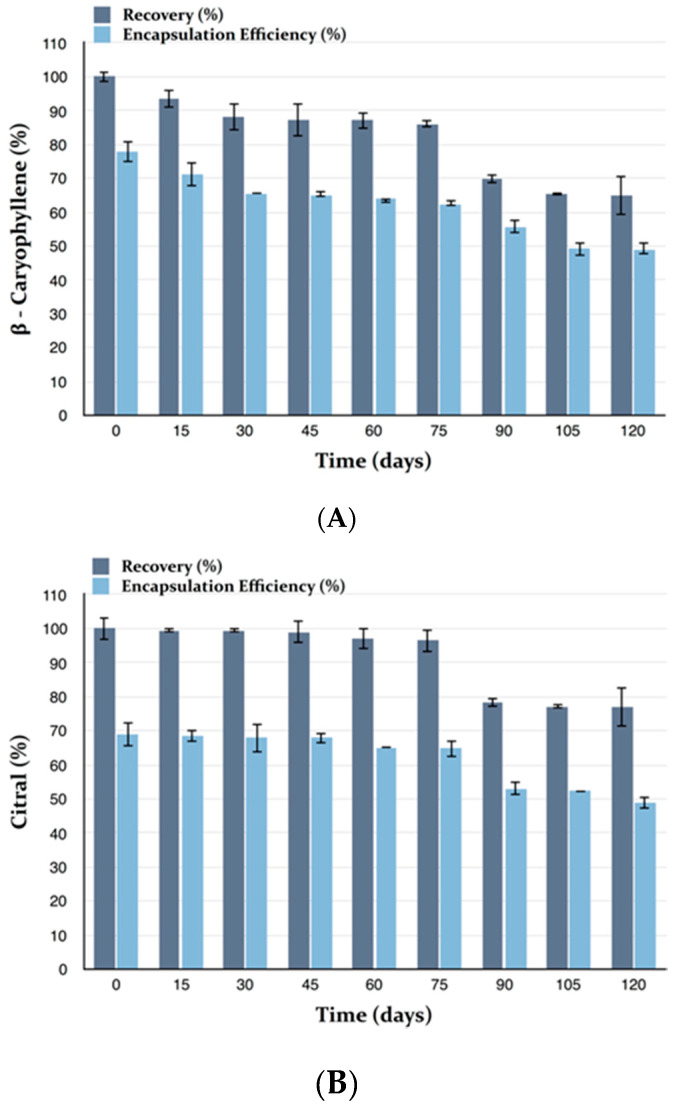
Chemical stability of *Melissa officinalis* essential oil-loaded glycerosomes (MEO-GS) during 4 months storage. Recovery (R) and encapsulation efficiency (EE) of MEO in terms of citral (**A**) and β-caryophyllene (**B**); (Mean ± SD; *n* = 3).

By contrast, after four months of storage of MEO-GS, citral concentration decreased by approximately 23% in terms of R and by 20% in terms of EE (Figure 5A). β-Caryophyllene concentration decreased by approximately 35% in terms of R, and by 29% in terms of EE (Figure 5B). According to the above results, both citral and β-caryophyllene reduction during storage is much less than that observed with the pure MEO, indicating that glycerosomes can better preserve these main MEO constituents from degradation processes or reduce their volatility.

### 2.7. Antiviral Assays Using Luciferase-Expressing HSV-1

**Figure 6:**
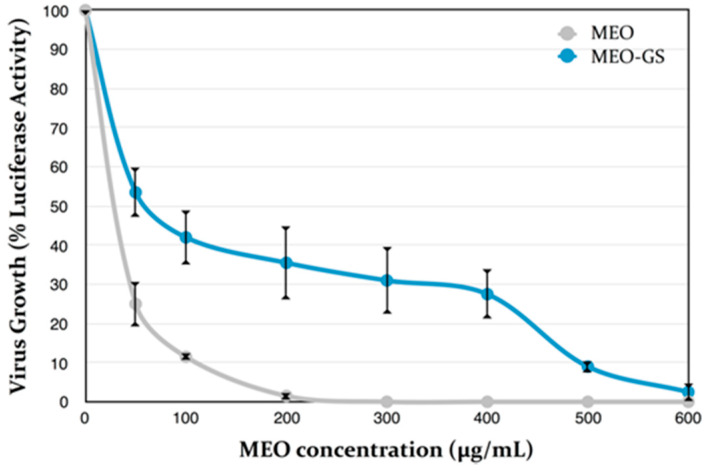
In vitro antiviral test. Effects of MEO and *Melissa officinalis* essential oil-loaded glycerosomes (MEO-GS) on the early steps of herpes simplex virus type 1 (HSV-1) infection; (Mean ± SD; *n* = 3).

**Figure 7:**
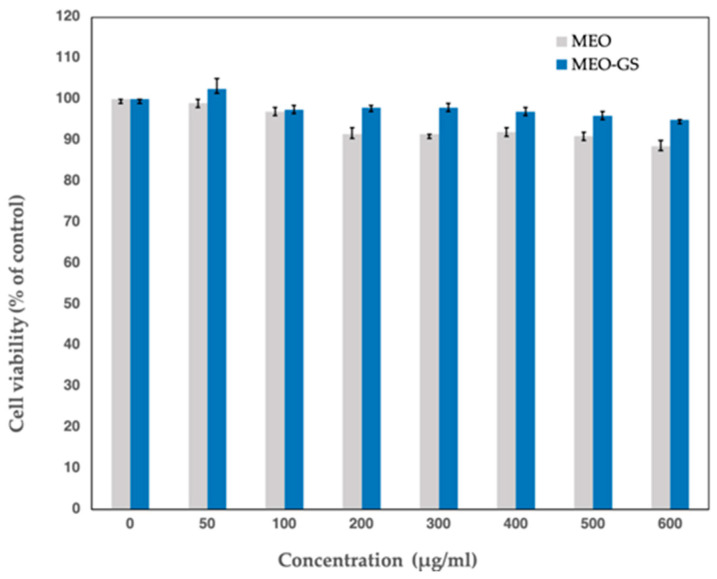
Cytotoxicity assays. Effects of short-term exposure (1 h) of Vero cells to *Melissa officinalis* essential oil-loaded glycerosomes (MEO-GS) and pure MEO on their viability; (Mean ± SD; *n* = 3).

Antiviral activity of MEO-GS and pure MEO was evaluated by in vitro assay on Vero cells. In order to accelerate the screening efforts for novel antivirals, the Department of Pharmacognosy/Pharmacology of the Aristotle University of Thessaloniki (Greece) generated an HSV-1 virus that expresses the firefly luciferase (LUC) gene under the control of the virus TK gene promoter/regulatory elements. This was achieved by substitution of the TK gene coding sequences with those of LUC. The resulting virus (vCLIDA61) expresses luciferase during the infection, with a linear relationship being observed between the amount of virus and the luciferase activity measured. The luciferase activity assays, which are easy, reproducible and very sensitive, can be used to assess virus growth and, by extrapolation, virus inhibition by antivirals, i.e., addition of an antiviral would reduce LUC expression in a dose-dependent manner. Exposure of the LUC-expressing virus (vCLIDA61) to increasing concentrations of MEO prior to and during the early stages of the infection (attachment, absorption and penetration) was found to inhibit LUC expression in a dose-dependent manner, as shown in Figure 6. The antiviral activity of MEO on cells already infected with HSV-1 could not be evaluated, since it was observed that prolonged exposure of cells to MEO produced cytotoxic effects, even at concentrations as low as 50 μg/mL. It must be noted that such cytotoxic effects were not observed upon short cell exposure (1 h) to either MEO or MEO-GS (Figure 7). The antiviral effects of glycerosomes containing MEO (MEO-GS), measured in parallel, were found to be less pronounced, except for with a high concentration of MEO, such as 500–600 µg/mL.

The results reported in the present study were similar, in terms of magnitude, to those of previous investigations. In particular, MEO inhibitory in vitro activity against HSV-1 and HSV-2 was reported on monkey kidney cells using the plaque reduction assay [13]. The IC_50_ values were 4 μg/mL and 0.8 μg/mL for HSV-1 and HSV-2, respectively. The toxic concentration for 50% of cells for HSV-1 was 30 μg/mL [13]. In a further study, MEO was tested on HSV-2 replication in HEp-2 cells. MEO was non-toxic to HEp-2 cells up to a concentration of 100 μg/mL. Strong anti-HVS-2 activity was found in the concentration range between 25 and 50 μg/mL [14]. In the literature, there are also a few studies concerning the activity of isolated constituents against HSV-1, namely citral and β-caryophyllene [6,7]. The maximum non-cytotoxic concentration of citral was 20 μg/mL, while the IC_50_ value was 23 μM, corresponding to about 6 μg/mL [6]. From the literature [7], β-caryophyllene showed a maximum non-cytotoxic concentration of 10 μg/mL when tested on HSV-1, while the cytotoxic concentration of the drug that reduced viable cell number by 50% was 35 μg/mL. IC_50_ was determined from dose-response curves as 0.25 μg/mL [7]. From the literature it is clear that native essential oils have higher selectivity indices rather than isolated constituents and are preferable for antiviral treatment in patients, however, citral and *β*-caryophyllene might be the dominant antiviral agents in MEO.

### 2.8. Cytotoxicity Assays

The potential cytotoxic effects of MEO-GS and MEO were evaluated by performing MTT assays. Specifically, Vero cells were exposed to increasing concentrations of MEO and MEO-GS for exactly 1 h, to mimic the conditions of exposure during the HSV-1 infection experiments. As shown in Figure 7, the observed cytotoxic effects of these short exposures to MEO and MEO-GS were minimal, even at the highest experimental concentrations (600 μg/mL).

## 3. Materials and Methods

### 3.1. Chemicals

Phosphatidylcholine (Phospholipon 90G, P90G) was purchased from Lipoid AG (Cologne, Germany) with the support of its Italian agent AVG srl (Milano, Italy). Cholesterol 95%, dichloromethane, methanol and acetonitrile were purchased from Sigma-Aldrich (Milan, Italy); vegetable glycerol Eur Ph. was purchased by Galeno srl (Prato, Italy). *Melissa officinalis* (lemon balm) essential oil was from Chiron Kentauros (Pelion, Greece). Ultrapure water was produced by a synergy UV Simplicity water purification system provided by Merck KGaA (Molsheim, France). Phosphotungstic acid (PTA) was purchased from Electron Microscopy Sciences (Hatfield, PA, USA).

### 3.2. Chemical Analysis of MEO by Gas Chromatography–Mass Spectrometry (GC–MS)

*Melissa officinalis* essential oil was analyzed by GC-MS, in order to identify the qualitative and quantitative composition. The analyses were performed by GC-2010-GCMS-QP2010 system (Shimadzu, Duisburg, Germany), operating at 70 eV. This was equipped with a split/splitless injector (230 °C) and a fused silica HP-5 MS capillary column (30 m × 0.25 mm i.d., film thickness 0.25 μm). The temperature program was from 50 to 290 °C, at a rate of 4 °C/min. Helium was used as a carrier gas at a flow rate of 1.0 mL/min. The injection volume of each sample was 1.0 μL. Arithmetic indices for all compounds were determined according to Van den Dool and Kratz [19], using n alkanes as standards. The identification of the components was based on comparison of their mass spectra with those of NIST21 and NIST107 [20], by comparison of their retention indices with literature data [21] and by co-chromatography with authentic constituents of MEO (Fluka, Sigma).

### 3.3. HPLC-DAD Analysis

Quantitative determination of MEO, in terms of citral and β-caryophyllene, the two main components of the essential oil, was carried out using the 1200 high performance liquid chromatograph (HPLC) equipped with a diode array detector (DAD), by Agilent Technologies Italia Spa (Rome, Italy). Chromatograms were acquired at 210 nm for β-caryophyllene and 233 nm for citral [22,23]. Chromatographic analyses were performed using a reverse-phase column Eclipse XDB C-18 (150 × 4.6) mm, 3.5 µm particle size, maintained at 27 °C. A gradient elution method, with 0.8 mL/min flow rate, was applied, using (A) acetonitrile and (B) formic acid/water (pH 3.2) as mobile phases. The analytical method was as follows: 0–3 min 80% (B), 3–20 min from 80% to 1% (B), 20–30 min 1% (B), 30–40 min 1–80% (B). The coefficient of determination (R^2^) was 0.9998 for citral calibration curve, and 0.9999 for β-caryophyllene calibration curve.

### 3.4. Preparation of Vesicles

MEO was loaded inside glycerosomes (MEO-GS), by the thin layer evaporation method in two steps [16], using a 10% *v*/*v* glycerol/water solution as medium. Different amounts of phosphatidylcholine (330 or 600 mg) and cholesterol (10 mg) were used and the experimental conditions of preparation were optimized by varying the hydration time, hydration volume and the optional use of an ultrasonication bath, as reported in Table 4. The selected formulation was prepared with 600 mg of phosphatidylcholine and 10 mg of cholesterol dissolved in dichloromethane, using the ultrasonication bath for 1 min, in order to improve their dissolution. Subsequently, evaporation of dichloromethane was carried out using rotavapor for 20 min at 30 °C, in order to obtain a homogenous lipid film on the internal surface of the flask. At this point, 100 μL of MEO was added inside the flask and the lipid film was hydrated with 5 mL of 10% glycerol/water solution, by using the mechanic stirrer [24] and the ultrasonication bath [25], for 30 min at 25 °C. Then, a further 5 mL of 10% *v*/*v* glycerol/water solution was added and the dispersion was mechanically shaken for a further 30 min at 25 °C, using an ultrasonication bath.

### 3.5. Physical Characterization of MEO-GS

Average hydrodynamic diameter (nm), polydispersity index (PdI) and ζ-potential (mV) of glycerosomes were measured by Dynamic and Electrophoretic Light Scattering, DLS-ELS (Zetasizer Nanoseries ZS90) by Malvern instrument (Worcestershire, UK) at 25 °C, with a scattering angle of 90 °C [26]. Glycerosomes were diluted using ultrapure water before measurements, in order to achieve a suitable scattering intensity. Successively, glycerosomes were observed by Transmission Electron Microscope, TEM (CM12 TEM, PHILIPS, Eindhoven, The Netherlands) equipped with an OLYMPUS Megaview G2 camera and with an accelerating voltage of 80 kV. A drop of sample, diluted 5-fold in water, was applied and dried by desiccation on a carbon film copper grid and it was counterstained with 1% (*w*/*v*) of phosphotungstic acid solution for 3 min. Then, the sample was examined at different amplifications.

### 3.6. Deformability

Deformability of glycerosomes was measured by extrusion, using the LipoFast-Basic extruder (Avestin Europe GmbH; Mannheim, Germany). The samples were extruded through a 19 mm polycarbonate membrane with 50 nm pore size (Avestin Europe GmbH; Mannheim, Germany), at a constant pressure of 7 bar, for 5 min. The extruded sample was collected in a syringe, meanwhile vesicle size and PdI were monitored by DLS analysis, before and after extrusion. Finally, deformability of vesicles was calculated according to the following Equation (1) [27]:

(1) D=average size (nm) before extrusion/average size (nm) after extrusion

### 3.7. Chemical Characterization of MEO-GS

Encapsulation efficiency (EE) and total recovery (R), of MEO inside glycersomes, were evaluated in terms of citral and β-caryophyllene, the two main components of the essential oil. EE was calculated according to the following Equation (2):

(2) $$EE=\left(\frac{encapsulated\;citral\;or\;\beta -caryophyllene\;}{initial\;citral\;or\;\beta -caryophyllene}\right)\;100$$

where *encapsulated citral or β-caryophyllene* is the concentration of the single components after the purification step. In fact, MEO-GS were purified from free MEO by the dialysis bag method [28], using Spectra/Por^®^ regenerated cellulose membranes with 3.5 KDa molecular weight cut-off (MWCO), by Repligen Europe B.V. (Breda, The Netherlands). The dialysis bag was stirred in 1 L of ultrapure water, at room temperature for 1 h. After that, the purified glycerosomes were diluted in methanol, in order to break vesicles and release the encapsulated MEO. Samples were centrifuged at 14,000 rpm for 10 min and they were analyzed by HPLC-DAD. MEO total recovery was determined using the same procedure without the purification step by dialysis, and it was calculated according to the following Equation (3):

(3) $$R=\left(\frac{total\;recovered\;citral\;or\;\beta -caryophyllene}{initial\;citral\;or\;\beta -caryophyllene}\right)\;100$$

where *total recovered citral or β-caryophyllene* is the concentration of the single components after the preparation procedure of MEO-GS.

### 3.8. In Vitro Release

The release of MEO from MEO-GS was evaluated by the dialysis bag method [29,30], using Spectra/Por^®^ regenerated cellulose membranes with 3.5 KDa MWCO), by Repligen Europe B.V. (Breda, The Netherlands), and it was compared to the release from a DMSO solution of MEO, at the same concentration of essential oil as used to prepared glycerosomes (10 mg/mL). The experiment was carried out with 1 mL of sample, magnetically stirred in 200 mL of PBS, used as release medium. The temperature was set at 37 °C; 0.5 mL of medium was collected at specified time points (30, 60, 120, 240, 360, 1440 min) and they were replaced by equal volumes of fresh medium, thus maintaining the sink conditions [31]. The collected release medium was analyzed by HPLC-DAD. The amount of released MEO was expressed as percentage of citral amount recovered in the release medium, as against citral amount contained inside the cellulose bag.

### 3.9. Stability Studies

Physical and chemical stability of MEO-GS were investigated after storing the samples 4 months at 4 °C away from light. Once every month, size, PdI and ζ-potential were measured by DLS-ELS, whereas R and EE of citral and β-caryophyllene were evaluated by HPLC-DAD. At the same storage conditions, MEO chemical stability was also monitored in terms of citral and β-caryophyllene concentration.

### 3.10. Cells, Viruses and Growth Conditions

In vitro assays were performed using African Green Monkey kidney cells (Vero), maintained in Dulbecco’s modified Eagle’s medium (DMEM), containing 10% fetal bovine calf serum (FCS), by Gibco BRL, Invitrogen, as described by Matta and coworkers [32]. The media were supplemented with 100 U/mL penicillin and 100 μg/mL streptomycin, provided by Sigma-Aldrich. The viruses were grown and titrated as previously described [33] and the virus titers were expressed in plaque-forming units (pfu) per mL. The HSV-1 strain vCLIDA61, used throughout the study, was generated at the Laboratory of Pharmacology of the Department of Pharmacognosy/Pharmacology, School of Pharmacy, Aristotle University of Thessaloniki, Thessaloniki, Greece. The virus was generated by substituting the thymidine kinase gene (TK, UL23) of the HSV ORF61 [34] with sequences encoding the firefly luciferase gene by homologous recombination. Upon infecting cells, vCLIDA61 expresses firefly luciferase under the control of the TK gene promoter/regulatory elements. Therefore, by measuring the luciferase activity it is possible to measure the virus growth and the progress of the HSV-1 infection. To measure the effects of MEO (free or glycerosome-formulated) on the subsequent steps of HSV-1 infection, i.e., post-entry, the Vero cell cultures were first infected with HSV-1 and then MEO or MEO-GS were added in the cell growth medium (DMEM supplemented with 10% FCS and antibiotics) at various concentrations.

### 3.11. Antiviral Assays

Vero cells were seeded into 12-well culture plates (Corning) at a density of 0.5 × 10^6^ cells/well and incubated at 37 °C with 5% CO_2_ until they reached 95% confluence. HSV-1 preparations (1500 plaque forming units (pfus) in 500 µL DMEM supplemented with 1% FCS) were pre-incubated for 60 min at 30 °C, either in the absence or presence of various concentrations of MEO (unformulated, dissolved in DMSO) or MEO-GS (glycerosome-formulated, as a diluted aqueous suspension), prior to being used to infect the Vero cell monolayers (100 μL virus preparation/well). After a 60-min infection of the cells at 37 °C the virus preparations were aspirated and DMEM, supplemented with 10% FCS and antibiotics, was added. The infection was allowed to proceed for 24 h prior to lysing the cells and assaying for luciferase activity. The above-described assays measure the effects of the essential oil both on the virus itself (virucidal activity) and on the early stages of virus infection (virus adsorption to and penetration of the target cells).

### 3.12. Luciferase Assays

The HSV-1-infected Vero cell monolayers, in the 12-well plates, were first washed with PBS before subsequently being lysed with 250 μL.

Luciferase lysis buffer (1% Triton X-100, 25 mM glycylglycine pH 7.8, 15 mM MgSO4, 4 mM EGTA, 1 mM dithiothreitol) for 7 min at room temperature. The luciferase activities were determined from triplicate infections, as previously described [35], using a Berthold Sirius luminometer. In short, luciferase assays were performed as follows: 100 μL from each lysate was placed in a reaction tube compatible with the luminometer (Sarstedt No 55.476, 75 × 12 mm) containing 500 μL luciferase (LUC) reaction buffer (25 mM glycylglycine pH 7.8, 15 mM MgSO_4_, 4 mM EGTA, 15 mM potassium phosphate, 1 mM dithiothreitol, 2 mM ATP). Each tube was then inserted into the luminometer, which had been programmed to inject 100 μL luciferin reagent (0.4 mM luciferin, 2 mM dithiothreitol, 25 mM glycylglycine pH 7.8, 15 mM MgSO_4_, 4 mM EGTA), and, after a 10-s delay, record light production (luciferase activity, in relative light units (rlu)) for 10 s at room temperature.

### 3.13. MTT Assay

The cytotoxicity assays were adapted from Armaka, et al. [36]. Specifically, Vero cells were seeded into 96-well plates at a density of 1 × 10^4^ cells/well in 100 μL DMEM supplemented with 5% FCS. Following a 1-h exposure to various MEO or MEO-GS concentrations, the cells were incubated for 48 h in fresh media before adding 10 μL of 5 mg/mL MTT and incubating for another 2.5 h at 37 °C. Following media removal, the blue formazan crystals formed by metabolically active live cells, were solubilized by shaking the plates for 1 h at 37 °C after the addition of 100 of solubilization solution (1 vol. 20% SDS, 1 vol. *N*,*N*-dimethylformamide, 5 vol. isopropanol) to each well, and the optical density was measured at 570 nm (test absorbance) and 630 nm (reference absorbance). The final value was obtained by subtracting the value obtained at 630 nm (nonspecific absorbance) from that obtained at 570 nm.

## 4. Conclusions

Glycerosomes are innovative type of vesicles, characterized by high stability and flexibility, with improved permeation through the skin and high in vitro biocompatibility toward human keratinocytes [16]. MEO-loaded glycerosomes (MEO-GS), developed in the present work, represent the first study related to the loading of glycerosomes with an essential oil for antiviral purposes. Our investigation proved that loaded-MEO did not modify the fluidity of the vesicle bi-layer, maintaining glycerosome ability to squeeze through skin pores. Developed glycerosomes are able to preserve MEO constituents from degradation processes during storage, as in the case with β-caryophyllene. In addition, the formulation has a strong anti-HSV-1 activity comparable to that of pure MEO, without cytotoxic effects. In conclusion, developed MEO-GS represent a potential strategic anti-herpetic tool to administer MEO, having numerous advantages over pure MEO, principally the preservation of the essential oil constituents and the extension of MEO release, once the formulation is applied on the skin.

## Figures and Tables

**Table 1:** *Melissa officinalis* essential oil (MEO) chemical composition obtained by Gas Chromatography–Mass Spectrometry (GC-MS) analysis. Data represent single compound percentages (%).

Constituents	%
1-Octen-3-ol	0.30
Methyl heptenone	1.88
Limonene	0.04
*cis*-Ocimene	0.05
*trans*-Ocimene	0.37
Linalool	0.51
*cis*-Rose oxide	0.11
exo-Isocitral	0.49
α-*trans*-Necrodol	0.56
Citronellal	4.31
(Ε)-Isocitral	1.75
4-*trans*-Caranone	2.62
Citronellol	0.19
Nerol	0.20
Neral	27.31
Geraniol	0.18
Methyl citronellate	0.28
Geranial	36.73
Methyl geranate	0.34
α-Copaene	0.15
β-Bourbonene	0.14
β-Cubebene	0.06
β-Elemene	0.14
β-Caryophyllene	14.85
α-Humulene	0.76
Germacrene D	1.55
α-Muurolene	0.05
γ-Cadinene	0.06
δ-Cadinene	0.14
Caryophyllene oxide	1.09
Total identified constituents	97.21

**Table 2:** Physical and chemical parameters of *Melissa officinalis* essential oil-loaded glycerosomes (MEO-GS). From left: Size, polydispersity index (PdI), ζ-potential, recovery (R) and encapsulation efficiency (EE); Mean ± SD (*n* = 3).

Sample	Size (nm)	PdI	ζ-potential (mV)	R (%)		EE (%)	
				**Citral**	**β-Car**	**Citral**	**β-Car**
MEO-GS *	83.09 ± 5.04	0.20 ± 0.05	−27.85 ± 4.03	73.80 ± 3.11	79.01 ± 8.71	51.27 ± 2.76	66.04 ± 8.76

* MEO-loaded glycerosomes.

**Table 3:** Deformability measurements of different formulations; (Mean ± SD; *n* = 3).

Sample	Size before Extrusion (nm)	Size after Extrusion (nm)	PdI before Extrusion	PdI after Extrusion	Deformability
MEO-GS *	83.92 ± 3.53	82.61 ± 2.56	0.25 ± 0.02	0.23 ± 0.01	1.02 ± 0.01
GS **	80.11 ± 6.92	79.68 ± 4.70	0.39 ± 0.04	0.36 ± 0.03	1.00 ± 0.03

* MEO-loaded glycerosomes; ** glycerosomes.

**Table 4:** Preparation of MEO-loaded glycerosomes (MEO-GS).

P90G:Chol Ratio (mg/mL)	MEO Conc (mg/mL)	Hydration Time (min)	Hydration Volume (mL)	Ultrasonication Bath
33:1	10	30	10	no
60:1	10	30	10	yes
60:1	10	30	10	no
60:1	10	30 + 30	5 + 5	yes
60:1	10	30 + 30	5 + 5	no
60:1	10	60	10	no
60:1	10	60 + 60	5 + 5	no

## Abbreviations

DAD	Diode Array Detector
DLS	Dynamic Light Scattering
DMEM	Dulbecco’s Modified Eagle’s Medium
ELS	Electrophoretic Light Scattering
EE	Encapsulation Efficiency
FCS	Fetal Bovine Calf Serum
GC–MS	Gas Chromatography–Mass Spectrometry
HSV	Herpes Simplex Virus
HPLC	High Performance Liquid Chromatograph
MEO	*Melissa officinalis* Essential Oil
MEO-GS	*Melissa officinalis* Essential Oil loaded in Glycerosomes
MTT	3-(4,5-dimethylthiazol-2-yl)-2,5-diphenyletrazolium bromide
NLC	Nanostructured Lipid Carrier
P90G	Phosphatidylcholine
PTA	Phosphotungstic acid
PdI	Polydispersity Index
R	Recovery
SLN	Solid Lipid Nanoparticles
TEM	Transmission Electron Microscopy
DMSO	Dimethyl sulfoxide

## References

[1] Whitley R., Baines J. (2018). Clinical management of herpes simplex virus infections: Past, present, and future. F1000 Res..

[2] Isacchi B., Fabbri V., Galeotti N., Bergonzi M.C., Karioti A., Ghelardini C., Vannucchi M.G., Bilia A.R. (2011). Salvianolic acid B and its liposomal formulations: Anti-hyperalgesic activity in the treatment of neuropathic pain. Eur. J. Pharm. Sci..

[3] Guccione C., Oufir M., Piazzini V., Eigenmann D.E., Jähne E.A., Zabela V., Faleschini M.T., Bergonzi M.C., Smiesko M., Hamburger M. (2017). Andrographolide-loaded nanoparticles for brain delivery: Formulation, characterisation and in vitro permeability using hCMEC/D3 cell line. Eur. J. Pharm. Biopharm..

[4] Isacchi B., Bergonzi M.C., Grazioso M., Righeschi C., Pietretti A., Severini C., Bilia A.R. (2012). Artemisinin and artemisinin plus curcumin liposomal formulations: Enhanced antimalarial efficacy against Plasmodium berghei-infected mice. Eur. J. Pharm. Biopharm..

[5] Schnitzler P. (2019). Essential Oils for the Treatment of Herpes Simplex Virus Infections. Chemotherapy.

[6] Astani A., Reichling J., Schnitzler P. (2010). Comparative Study on the Antiviral Activity of Selected Monoterpenes Derived from Essential Oils. Phytother. Res..

[7] Astani A., Reichling J., Schnitzler P. (2011). Screening for antiviral activities of isolated compounds from essential oils. Evid Based Complement Altern. Med..

[8] European Scientific Cooperative on Phytotherapy (ESCOP) (2013). Melissae folium, *Melissa officinalis* L. leaf. ESCOP Monograph.

[9] Astani A., Heidary Navid M., Schnitzler P. (2014). Attachment and Penetration of Acyclovir-resistant Herpes Simplex Virus are inhibited by *Melissa officinalis* Extract. Phytother. Res..

[10] Astani A., Reichling J., Schnitzler P. (2012). Melissa officinalis extract inhibits attachment of herpes simplex virus in vitro. Chemotherapy.

[11] Mazzanti G., Battinelli L., Pompeo C., Serrilli A.M., Rossi R., Sauzullo I., Vullo V. (2008). Inhibitory activity of Melissa officinalis L. extract on Herpes simplex virus type 2 replication. Nat. Prod. Res..

[12] Nolkemper S., Reichling J., Stintzing F.C., Carle R., Schnitzler P. (2006). Antiviral effect of aqueous extracts from species of the Lamiaceae family against Herpes simplex virus type 1 and type 2 in vitro. Planta Med..

[13] Schnitzler P., Schuhmacher A., Astani A., Reichling J. (2008). *Melissa officinalis* oil affects infectivity of enveloped herpesviruses. Phytomedicine.

[14] Allahverdiyev A., Duran N., Ozguven M., Koltas S. (2004). Antiviral activity of the volatile oils of *Melissa officinalis* L. against Herpes simplex virus type-2. Phytomedicine.

[15] Bilia A.R., Guccione C., Isacchi B., Righeschi C., Firenzuoli F., Bergonzi M.C. (2014). Essential oils loaded in nanosystems: A developing strategy for a successful therapeutic approach. Evid Based Complement Altern. Med..

[16] Manca M.L., Zaru M., Manconi M., Lai F., Valenti D., Sinico C., Fadda A.M. (2013). Glycerosomes: A new tool for effective dermal and transdermal drug delivery. Int. J. Pharm..

[17] de Matos S.P., Teixeira H.F., de Lima Á.A., Veiga-Junior V.F., Koester L.S. (2019). Essential oils and isolated terpenes in nanosystems designed for topical administration: A review. Biomolecules.

[18] Jain S., Jain P., Umamaheshwari R.B., Jain N.K. (2003). Transfersomes—a novel vesicular carrier for enhanced transdermal delivery: Development, characterization, and performance evaluation. Drug Dev. Ind. Pharm..

[19] Van den Dool H., Kratz P.D. (1963). A generalization of the retention index system including linear temperature programmed gas-liquid partition chromatography. J. Chromatogr. A.

[20] Masada Y. (1976). Analysis of Essential Oils by Gas Chromatography and Mass Spectrometry.

[21] Adams R.P. (2007). Identification of Essential Oil Components by Gas Chromatography/Mass Spectrometry.

[22] de Almeida Borges V.R., Ribeiro A.F., de Souza Anselmo C., Cabral L.M., de Sousa V.P. (2013). Development of a high performance liquid chromatography method for quantification of isomers β-caryophyllene and α-humulene in copaiba oleoresin using the Box-Behnken design. J. Chromatogr. B.

[23] Gaonkar R., Yallappa S., Dhananjaya B.L., Hegde G. (2016). Development and validation of reverse phase high performance liquid chromatography for citral analysis from essential oils. J. Chromatogr. B.

[24] van Hoogevest P. (2017). Review–An update on the use of oral phospholipid excipients. Eur. J. Pharm. Sci..

[25] Zhang K., Zhang Y., Li Z., Li N., Feng N. (2017). Essential oil-mediated glycerosomes increase transdermal paeoniflorin delivery: Optimization, characterization, and evaluation in vitro and in vivo. Int. J. Nanomed..

[26] Bhattacharjee S. (2016). DLS and zeta potential–what they are and what they are not?. J. Control. Release.

[27] Vanti G., Bani D., Salvatici M.C., Bergonzi M.C., Bilia A.R. (2019). Development and percutaneous permeation study of escinosomes, escin-based nanovesicles loaded with berberine chloride. Pharmaceutics.

[28] Bilia A.R., Nardiello P., Piazzini V., Leri M., Bergonzi M.C., Bucciantini M., Casamenti F. (2019). Successful Brain Delivery of Andrographolide Loaded in Human Albumin Nanoparticles to TgCRND8 Mice, an Alzheimer’s disease Mouse Model. Front Pharmacol..

[29] Moreno-Bautista G., Tam K.C. (2011). Evaluation of dialysis membrane process for quantifying the in vitro drug-release from colloidal drug carriers. Colloids Surf. A Physicochem. Eng. Asp..

[30] Risaliti L., Kehagia A., Daoultzi E., Lazari D., Bergonzi M.C., Vergkizi-Nikolakaki S., Hadjipavlou-Litina D., Bilia A.R. (2019). Liposomes loaded with *Salvia triloba* and *Rosmarinus officinalis* essential oils: In vitro assessment of antioxidant, antiinflammatory and antibacterial activities. J. Drug Deliv. Sci. Technol..

[31] Asprea M., Tatini F., Piazzini V., Rossi F., Bergonzi M.C., Bilia A.R. (2019). Stable, monodisperse, and highly cell-permeating nanocochleates from natural soy lecithin liposomes. Pharmaceutics.

[32] Matta M.K., Panagiotidis C.A. (2008). High-mobility group protein A1 binds herpes simplex virus gene regulatory sequences and affects their expression. Arch. Virol..

[33] Nishioka Y., Silverstein S. (1977). Degradation of cellular mRNA during infection by herpes simplex virus. Proc. Natl. Acad. Sci. USA.

[34] Kyratsous C.A., Walters M.S., Panagiotidis C.A., Silverstein S.J. (2009). Complementation of a herpes simplex virus ICP0 null mutant by varicella-zoster virus ORF61p. J. Virol..

[35] Panagiotidis C.A., Silverstein S.J. (1999). The host-cell architectural protein HMG I (Y) modulates binding of herpes simplex virus type 1 ICP4 to its cognate promoter. Virology.

[36] Armaka M., Papanikolaou E., Sivropoulou A., Arsenakis M. (1999). Antiviral properties of isoborneol, a potent inhibitor of herpes simplex virus type 1. Antivir. Res..

